# Closed Keloid Therapy Supplying Water (by Gel) and Avoiding Air (by Being Wrapped With PERMEROLL): A Case Report

**DOI:** 10.1155/crdm/1258930

**Published:** 2025-04-28

**Authors:** Haruyoshi Yamada

**Affiliations:** Yamada Dermatologic Clinic, 3-1-21 Nishikatakura, Hachiouji-shi, Tokyo, Japan

## Abstract

Keloids and hypertrophic scars share the same pathology, with immoderate collagen production being their main characteristic. Moreover, keloids and hypertrophic scars may occur as sequential disorders. Many studies have developed methods for treating these diseases; however, none have become the golden standard treatment. In this case study, closed keloid therapy using PERMEROLL (Nitoms, Inc., Tokyo, Japan) was administered to a 71-year-old female who presented with bacillus Calmette–Guerin keloids on her shoulder that had persisted for approximately 60 years. The gel (ITO Co., Ltd., Tokyo, Japan) was attached to the lesion, and the keloidal lesion was covered with PERMEROLL. Ten months following treatment, the hardness of the lesion, especially at its centre, had softened, its colour had lightened compared with the periphery and the thickness of the keloidal lesion had decreased. Overall, satisfactory results were noted. Closed keloid therapy with PERMEROLL enables the complete enclosure of the lesion and is an easier and cheaper method than the former treatment agent. Moreover, decreasing the level of thymic stromal lymphopoietin may be crucial for treating keloids.

## 1. Introduction

Keloids and hypertrophic scars share the same pathology, with immoderate collagen production being their main characteristic. Hypertrophic scars do not develop beyond the margins of the initial wound, while keloids grow beyond the borders of the initial wound [1–3]. Keloids and hypertrophic scars appear to be sequential fibroproliferative diseases [4].

Trehalose, a disaccharide with a moisturising effect, can reportedly reduce thymic stromal lymphopoietin (TSLP) mRNA expression levels and consequently reduce transforming growth factor beta (TGF-β) and collagen deposition [5, 6].

Trehalose, with a molecular weight of 342 g/mol, can penetrate the epidermis and be dissolved in a gel that is applied to the keloid-affected skin surface. However, based on a previous controlled study by Yamada, applying the gel without trehalose led to good results in some patients [7].

In this case study, gel without trehalose was applied to the keloidal surface and then wrapped with PERMEROLL. The common treatment for keloids currently includes the application of steroid ointments, steroid tape or both as the first-line treatment. Herein, we present the case of a patient who received keloid treatment using a specialised dressing called PERMEROLL (Nitoms Inc., Tokyo, Japan).

## 2. Case Presentation

**Figure 1:**
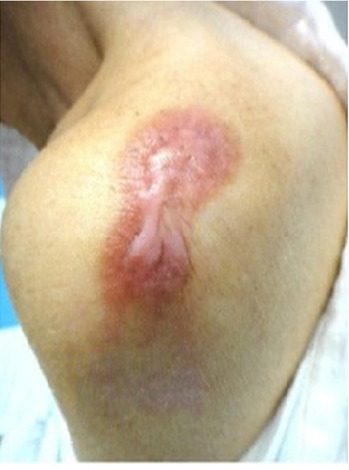
Pretreatment of a 71-year-old female patient presenting with a bacillus Calmette–Guerin keloid on her left shoulder that had persisted for approximately 60 years.

A 71-year-old female patient presented with a bacillus Calmette–Guerin keloid on her shoulder that had persisted for approximately 60 years. Slight pruritus and tingling had been noted for a few years, and the keloid was red, firm and raised and had an irregular shape. The patient provided written informed consent for the publication of the photographs depicting the lesions, and the requirement of ethics review board approval was waived owing to the nature of the study. The keloid was treated using an ITO gel base, and PERMEROLL was subsequently applied (Figure 1). A small amount of gel (approximately one-half of a fingertip) was applied daily to the keloid, which was subsequently wrapped.

The gel (ITO gel base; ITO Co., Ltd., Tokyo, Japan) comprised water, glycerin, carboxy acrylic polymer, sodium polyacrylate, methylparaben and propylparaben. The wrapping film (PERMEROLL; Nitoms, Inc.) comprised a waterproof but highly water vapour-permeable soft gel adhesive.

Throughout the trial, no other treatments, such as a pressure garment, steroid ointment application or intralesional steroid injection, were administered. During this period, the patient visited the clinic every 1-2 months.

In the outpatient clinic, at least 50 patients who had scars (i.e., hypertrophic and keloidal) were treated using the gel-PERMEROLL method. A few patients had mild contact dermatitis due to the gel or PERMEROLL. However, no severe side effects have occurred to date.

**Figure 2:**
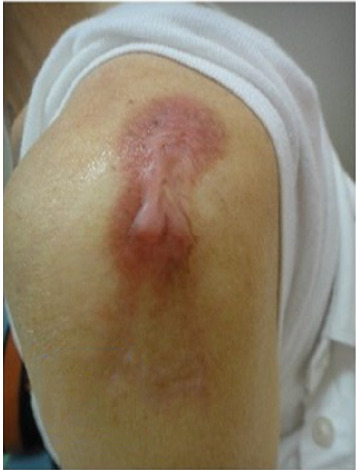
Four months following treatment. The patient was treated with closed keloid therapy using PERMEROLL (Nitoms, Inc., Tokyo Japan). Four months following treatment, although still hard, the keloid was softer than at the time of the initial presentation.

**Figure 3:**
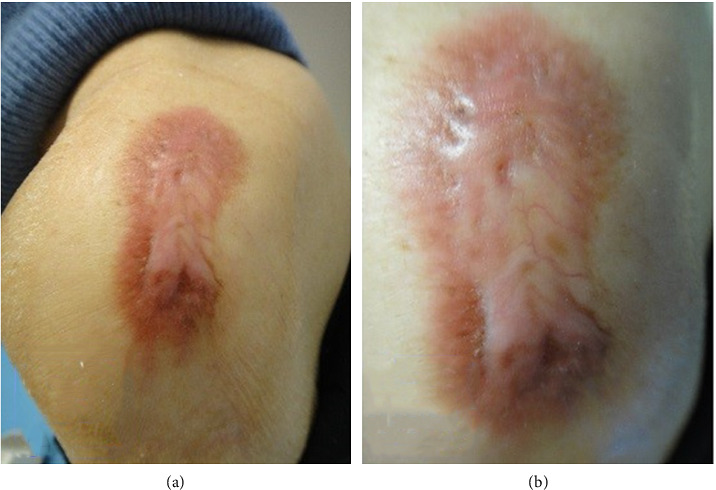
Ten months following treatment. (a) The same bacillus Calmette–Guerin keloid on the shoulder of the said 71-year-old female, 10 months following treatment. (b) The high-magnification of the clinical feature.

After 1 month of treatment, the keloid became moderately hard, and pruritus occurred less frequently, although the keloid's appearance was unchanged. Four months following treatment, although still hard, the keloid was softer than that during the initial presentation (Figure 2). The patient experienced no tingling but reported mild pruritus and pain in the keloid upon exposure to cold 6 months following treatment. Ten months following treatment, the colour of the keloid had changed from dark brown-red to light brown-red (Figures 3(a) and 3(b)).

The keloid's hardness had been decreasing, and the centre of the lesion was softer and lighter than the border area. In addition, its thickness was decreasing. I believe that closed keloid therapy can yield similar or even more favourable results than those achieved through steroidal tape attachment.

## 3. Discussion

This report presents the case of a keloid that had persisted for 60 years and was successfully treated using closed keloid therapy with PERMEROLL. Hydration and occlusion for hypertrophic scars and keloids have previously been reported as important treatment mechanisms [8], and the use of water-containing gels, in addition to a hard plastic film covering, has been promoted [8].

Moreover, occlusive conditions surrounding the lesion may have increased the effectiveness of treatment in our patient. In a previous case report, a mechanism of hydration and hypoxic-occlusion treatment for keloids and hypertrophic scars was proposed using gel and polyethylene food wrap [7]. In the present case, PERMEROLL was used. Water vapour can permeate through PERMEROLL; however, it remains waterproof.

In this patient, the collagen deposition of the keloid was reduced (Figures 1, 3(a), and 3(b)), as evidenced by the decreased thickness of the lesion on palpation. At the molecular level, the hypoxic-occlusive conditions created using PERMEROLL can induce the expression of hypoxia-inducible factor-2 (HIF-2). Increased HIF-2 expression levels downregulate TSLP, thereby reducing TGF-β levels and collagen deposition [6, 9–11].

This case study had some limitations. First, the biological membrane moves in multiple directions, allowing inflamed skin and scars to come into contact with more oxygen from the outer skin surface (room air) than the normal skin. This is owing to the incomplete skin barrier [12]. However, the various levels of tissue oxygenation could not be specifically examined. Future studies on the treatment of scars with PERMEROLL alone to discern whether gel application is needed are warranted.

Second, oxygen saturation is a relative measure of the concentration of oxygen that is dissolved and can differ among individuals, depending on a lesion's location [11]. Thus, analysing gene expression in keloidal fibroblasts under hypoxic conditions is necessary.

## 4. Conclusion

This study showed the successful treatment of a keloid using a gel and specialised dressing. Depriving the keloid surface of air is essential for its treatment. Reducing TSLP levels by covering the keloid surface with PERMEROLL is also the key for successful treatment.

## Data Availability

All data supporting the results are available as part of the article and no additional source data are required.
